# Genomic Analysis Provides New Insights Into Biotechnological and Industrial Potential of *Parageobacillus thermantarcticus* M1

**DOI:** 10.3389/fmicb.2022.923038

**Published:** 2022-06-09

**Authors:** Songul Yasar Yildiz, Ilaria Finore, Luigi Leone, Ida Romano, Licia Lama, Ceyda Kasavi, Barbara Nicolaus, Ebru Toksoy Oner, Annarita Poli

**Affiliations:** ^1^Department of Bioengineering, Istanbul Medeniyet University, Istanbul, Turkey; ^2^Institute of Biomolecular Chemistry (ICB), National Research Council, Naples, Italy; ^3^Department of Bioengineering, Industrial Biotechnology and Systems Biology (IBSB), Marmara University, Istanbul, Turkey

**Keywords:** *Parageobacillus thermantarcticus*, thermophiles, genome, next-generation sequencing, exopolysaccharides

## Abstract

*Parageobacillus thermantarcticus* strain M1 is a Gram-positive, motile, facultative anaerobic, spore forming, and thermophilic bacterium, isolated from geothermal soil of the crater of Mount Melbourne (74°22′ S, 164°40′ E) during the Italian Antarctic Expedition occurred in Austral summer 1986–1987. Strain M1 demonstrated great biotechnological and industrial potential owing to its ability to produce exopolysaccharides (EPSs), ethanol and thermostable extracellular enzymes, such as an xylanase and a β-xylosidase, and intracellular ones, such as xylose/glucose isomerase and protease. Furthermore, recent studies revealed its high potential in green chemistry due to its use in residual biomass transformation/valorization and as an appropriate model for microbial astrobiology studies. In the present study, using a systems-based approach, genomic analysis of *P. thermantarcticus* M1 was carried out to enlighten its functional characteristics. The elucidation of whole-genome organization of this thermophilic cell factory increased our understanding of biological mechanisms and pathways, by providing valuable information on the essential genes related to the biosynthesis of nucleotide sugar precursors, monosaccharide unit assembly, as well as the production of EPSs and ethanol. In addition, gene prediction and genome annotation studies identified genes encoding xylanolytic enzymes that are required for the conversion of lignocellulosic materials to high-value added molecules. Our findings pointed out the significant potential of strain M1 in various biotechnological and industrial applications considering its capacity to produce EPSs, ethanol and thermostable enzymes *via* the utilization of lignocellulosic waste materials.

## Introduction

*Parageobacillus thermantarcticus* strain M1 (DSM 9572*^T^*) is a thermophilic rod-shaped bacterium isolated from geothermal soil of the crater of Mount Melbourne (74°22′ S, 164°40′ E) during the Italian Antarctic Expedition in Austral summer of 1986–1987 (Nicolaus et al., 1996).

The taxonomical assignment of strain M1 underwent several changes over the years. Originally, at the time of the first taxonomic description, Nicolaus et al. (1996) indicated it by the name of *Bacillus thermoantarcticus*, which was subsequently corrected to “*thermantarcticus*” in the Validation List N. 84 published in IJSEM (Finore et al., 2019). Based on the 16S rRNA gene sequence analysis, strain M1 was placed as a peripheral member of the thermophilic *Bacillus* rRNA group 5. Afterward, Coorevits et al. (2012) illustrated the phenotypic and phylogenetic distance of strain M1 from other *Bacillus* species and transferred *B. thermantarcticus* to the genus *Geobacillus* as *Geobacillus thermantarcticus* comb. nov.

When the whole genome sequences of strain M1 and other *Geobacillus* species became available, Aliyu et al. (2016) analyzed the phylogenetic relatedness, applying the Average Amino acid Identity, the Average Nucleotide Identity and the digital DNA-DNA hybridization. Phylogenomic comparisons indicated that the genus *Geobacillus* clustered into two monophyletic clades that differed in terms of nucleotide base composition. Considering this premise, it was found that the *Geobacillus* species (comprising *G. thermantarcticus*), currently residing within clade II, were reconsidered as belonging to a new genus named *Parageobacillus*.

Several interesting enzymatic pathways were found in *P. thermantarcticus*, making this Antarctic species of great potential in biotechnological applications, ranging from bioprocessing tools to industrial biocatalysis. Thermostable enzymatic activities were studied, such as an intracellular xylose/glucose isomerase and a protease, as well as an extracellular xylanase (1,4-β-D-xylanxylanohydrolase; E.C. 3.2.1.8) and a β-xylosidase (1,4-β-D-xylanxylohydrolase; E.C. 3.2.1.37) (Finore et al., 2019). Presence of xylanolytic enzymes was exploited to develop and optimize processes for the conversion of lignocellulosic biomass into high value added molecules like monosaccharides and valuable prebiotics (xylooligosaccharides) (Lama et al., 2001; Finore et al., 2016). In particular, enzymatic conversions involved the hemicellulolytic fractions extracted by eco-sustainable processes from the stems and leaves of *Cynara cardunculus* and from rhizome biomass of *Arundo donax*, where only the seeds of the former and the stems of the latter are employed for bioethanol production when the enzymatic performances are compared with those described for the other *Bacillus* species, *P. thermantarcticus* enzymes stand out with remarkable optimum working conditions (80°C and pH 5.6 for xylanase and 70°C and pH 6.0 for β-xylosidase). Moreover, during the late exponential phase of growth, *P. thermantarcticus* was able to synthesize cytoplasmatic D-xylose (glucose) isomerase, which transforms both xylose and glucose into xylulose and fructose, respectively, and was remarkably stable at high temperatures (optimal temperature 90°C, pH 7.0) (Lama et al., 2001). Different carbon sources were tested to improve the enzyme production and the best yield was obtained when xylose was employed as unique substrate in the growth media, both for xylose isomerase and glucose–isomerase activities.

Dipasquale et al. (2008) reported the presence of an extracellular proteolytic enzyme in *P. thermantarcticus* and its maximum yield was detected at the end of the exponential growth phase. Skim milk in the growth medium was found to enhance the protease production up to 19-fold.

Besides the biotechnological potentials associated with these thermostable enzymes, successful studies have also demonstrated its employment as a suitable microbial model for Astrobiology, i.e., the multidisciplinary approach to the study of origin and evolution of life on Earth and in the Universe. Several experiments were carried out either in space or in laboratories, by simulating the extreme environmental parameters that are typical of space and exoplanets, such as irradiation with UV and γ-rays, desiccation, low temperature values, etc. (Di Donato et al., 2018b; Romano et al., 2018, 2022; Camerlingo et al., 2020). *P. thermantarcticus* viable cells and its spores were found to survive in a space simulated environment, which in turn lead to a new scenario called as the Panspermia theory that states that life on Earth could have originated from bacterial species from other places transported by solar pressure.

The Antarctic cell factory strain M1 was also studied for its ability to produce two exopolysaccharides (EPSs) using mannose as a sole carbon and energy source, with a yield of 400 mg/L, and spectroscopic analyses have revealed their chemical nature as xanthan and mannan-type polymers (Manca et al., 1996). As with other EPS producing thermophiles, these biopolymers represent an ecological strategy to resist the extreme conditions: due to their protective nature, EPSs, surrounding the cells, represent a defense mechanism against extreme values of temperature and salinity; they are essential in retention of nutrients of water (Poli et al., 2017; Di Donato et al., 2018a). Without any doubt, EPSs are rapidly emerging as new and industrially important biomaterials, essentially thanks to their unique and complex chemical structures and properties, with commercial applications in various fields, from agriculture to medicine, microbial enhanced oil recovery (MEOR) and wastewater treatment, passing through the food and pharmaceutical fields, as well as packaging, textile and cosmetics use. Another important advantage is their high degree of their biocompatibility, biodegradability and both environmental and human compatibility, which make them viable alternatives to petroleum-based polymers (Ates, 2015). Despite the excellent qualities of microbial EPSs, only a few have achieved commercial use due to their high production costs. The interrelations between metabolic pathways and EPS biosynthesis need to be elucidated in order to enhance the microbial productivity, and it is the mandatory step for the maximum exploitation and use of these biopolymers (Wang et al., 2019). Therefore, a system-based approach becomes essential to pursue the objectives of industrial applications and for understanding the interplay between metabolism and bacterial EPS biosynthesis. Since microbial EPS biosynthesis is a result of a complex system of many metabolic pathways, the interplay of these networks needs to be enlightened to control, improve chemical characteristics and optimize production in order to achieve the formerly reported yields. In the view of this, the whole genome of M1 strain was sequenced^1^ by the U.S. Department of Energy Joint Genome Institute, *via* Illumina HiSeq 2500-1TB, and the NCBI Bioproject Accession number PRJNA323262 was assigned. For the data. The total number of sequenced bases was 3,448,881, of which 85.19% were coding bases and the DNA C + G% was found to be 43.63%. The total number of genes was 3,714, of which 96.85% were protein-coding and the remaining 3.15% were RNA genes. The number of protein-coding genes with function prediction was 2,783 (79.93%), while the number of those without function prediction were 814.

The genome sequencing and annotation can be a potent tool for understanding the genes utilized for biopolymer production. Moreover, elucidation of EPS biosynthetic pathways could lead to metabolic and genomic engineering strategies for the overproduction and tailor-made synthesis of biopolymers with desired activities and properties.

Hence in the present study, genome analysis of *P. thermantarcticus* M1 was performed with the aim of understanding the real potential of this organism. Elucidation of the whole genome organization of this thermophilic cell factory increased our understanding of biological mechanisms and pathways, by providing valuable information on the essential genes related to the biosynthesis of nucleotide sugar precursors, monosaccharide unit assembly, as well as the production of EPSs and ethanol. Presence of genes encoding xylanolytic enzymes that are required for the hydrolysis of hemicellulose indicated the potential use of this organism in the production of high-value added industrial products, such as ethanol, from lignocellulosic biomass. Our findings pointed out the significant potential of strain M1 in various biotechnological and industrial applications, considering its capacity to produce EPSs, ethanol and thermostable enzymes via the utilization of lignocellulosic waste materials.

## Materials and Methods

### Bacterial Strain and Culture Conditions

*P. thermantarcticus* (DSM 9572*^T^*) strain M1 (Nicolaus et al., 1996), a facultative anaerobic thermophilic Gram-positive bacterium, isolated from geothermal soil of Mount Melbourne in Antarctica, was cultured in a flask at 60°C for 12 h. Cells were grown in YN standard complex medium containing 6.0 g/L yeast extract and 3.0 g/L NaCl at pH 6.0 (Lama et al., 2004; Finore et al., 2019). For the DNA analysis, the cells of strains M1 were collected at the exponential phase, *via* centrifugation at 5,000 × g for 15 min. Genomic DNA was isolated using DNAzol (Molecular Research Center, Inc., Cincinnati, OH, United States) according to the manufacturer’s instructions. The DNA was quantified spectrophotometrically using a Qubit™ fluorimeter (Thermo Fisher Scientific, MA, United States). The amount of 5 μg/μl of gDNA was considered for the construction of Illumina 300 bp library. The genome was sequenced at Department of Energy Joint Genome Institute under the project entitled: “The Genomic Encyclopedia of Bacteria and Archaea (GEBA).”

### Whole Genome Sequence and Genome Assembly

The whole genome of *P. thermantarcticus* strain M1 was sequenced *via* Illumina HiSeq 2000 1TB technology. The library method was Illumina Regular Fragment, 300 bp, Plates with insert sizes of 238 ± 34 bp. The genome was assembled from a total of 6,360,534 sequence reads, with an average read length of 151 bp. The reads were assembled into 106 contigs with an average contig length of about 32,536 bp.

### Genome Annotation

Genome annotation and gene prediction of *P. thermantarcticus* M1 were carried out using auto-annotation servers, such as Rapid Annotations using Subsystems Technology (RAST),^2^ and Pathosystems Resource Integration Center (PATRIC).^3^ The results obtained from the RAST Server was controlled by matching to that from PATRIC. The predictions of protein-encoding, rRNA and tRNA genes were made by RAST Server. The predictions of genes involved in important subsystems were manually verified *via* BLASTp^4^ against protein databases (i) the Universal Protein Resource (UniProt)^5^ and (ii) National Center for Biotechnology Information (NCBI).^6^ Subsystem annotation obtained from the RAST Server was used for function and category assignments to genes in the genome. Information on genes that encode enzymes was attained from Kyoto Encyclopedia of Genes and Genomes (KEGG)^7^ and ExPASy^8^ databases.

### Phylogenomic Analysis

Phylogenomic comparison was performed between the whole genome sequence (WGS) of *P. thermantarcticus* M1 (GenBank accession number FOJS00000000.1), and WGS of the other species of the *Parageobacillus* genus [*P. thermoglucosidasius* DSM 2542 (GenBank accession number NZ_CP012712.1), *P. toebii* DSM 14590 (GenBank accession number RCWX00000000.1), *P. caldoxylosilyticus* CIC9 (GenBank accession number AMRO00000000.1), *P. genome* sp.1 NUB3621 2542 (GenBank accession number AOTZ00000000.1)]. NCBI database was used to download the WGS of the species and MicroScope Microbial Genome Annotation and Analysis Platform^9^ was used for the comparison of the genomes. MicroScope Microbial Genome Annotation and Analysis Platform assigned putative functions to the proteins encoded and these functions were used in order to compare the selected genomes. Comparison was performed with permissive parameters (50% amino-acid identity, 80% amino-acid alignment coverage).

### Comparative Genomics

WGS of 18 strains belonging to 5 different *Parageobacillus* species [*P. thermantarcticus* M1 (GenBank accession number FOJS00000000.1), *P. thermoglucosidasius* ZCTH02-B4 (GenBank accession number LZRS00000000.1), *P. thermoglucosidasius* DSM 2542 (GenBank accession number NZ_CP012712.1), *P. thermoglucosidasius* C56-YS93 (GenBank accession numbers of chromosome CP002835.1 and plasmids CP002836.1/CP002837.1), *P. thermoglucosidasius* NCIMB 11955 (GenBank accession numbers of chromosome CP016622.1 and plasmids CP016623.1/CP016624.1), *P. thermoglucosidasius* TM242 (GenBank accession numbers of chromosome CP016916.1 and plasmids CP016917.1/CP016918.1), *P. thermoglucosidasius* TNO-09.020 (GenBank accession number CM001483.1), *P. thermoglucosidasius* NBRC 107763 (GenBank accession number BAWP00000000.1), *P. thermoglucosidasius* W-2 (GenBank accession number LXMA00000000.1), *P. thermoglucosidasius* GT23 (GenBank accession number LUCT00000000.1), *P. toebii* NBRC 107807 (GenBank accession number BDAQ00000000.1), *P. toebii* DSM 14590 (GenBank accession number RCWX00000000.1), *P. toebii* B4110 (GenBank accession number LQYW00000000.1), *P. toebii* PW12 (GenBank accession number QREZ00000000.1), *P. caldoxylosilyticus* NBRC 107762 (GenBank accession number BAWO00000000.1), *P. caldoxylosilyticus* CIC9 (GenBank accession number AMRO00000000.1), *P. caldoxylosilyticus* B4119 (GenBank accession number LQYS00000000.1), *P. genomosp.1* NUB3621 2542 (GenBank accession number AOTZ00000000.1)] were obtained from the NCBI database and annotated automatically by the RAST Server and PATRIC. Encoding proteins that played roles in carbohydrate utilization, and industrially or biotechnologically important pathways, such as xylan hydrolysis, ethanol and, EPS biosynthesis, were found out for 4 genomes of different species and comparative analysis was performed according to the presence/absence of proteins.

### Exopolymer Production and Recovery

The precultures of *P. thermantarcticus* strain M1 were incubated in YN standard complex medium for 8 h; then 100 ml were used to inoculate the culture. The cells were grown in Biostat C-plus Fermenter 10–3 (Sartorius Stedim; Melsungen, Germany) with a working volume of 4 L at 60°C with an agitation speed of 150 rpm. The fermentations were carried out under aerobic conditions with a constant air flow of 1.5 L min^–1^. pH was adjusted to 6.5 with 2M H_2_SO_4_ and 1M NaOH. Fermentation medium containing 0.6% (w/v) carbon source, 0.1% (w/v) yeast extract, and 0.3% (w/v) NaCl, was sterilized *in situ* at 120°C for 20 min, except that carbon source solution which was added to a sterile medium after filtration (0.22 μm size-pore). Cells were grown on various carbon sources, including fructose, galactose, maltose, xylose, mannose, trehalose, glycerol, lactose, glucose, sucrose. Growth was monitored by measuring the absorbance at 540 nm until the stationary phase was reached.

Samples taken from the cultures were centrifuged at 10,000 rpm for 30 min at 4°C. Equal volume of cold absolute ethanol was added to the polymeric substances in the extracellular fraction and kept at −20°C overnight. In order to precipitate exopolymer, the mixture was centrifuged for 30 min at 10,000 rpm at 4°C. Warm distilled water was used to dissolve the pellet and it was dialyzed against water in dialysis tubes (Spectra/Por MWCO; molecular weight cut-off 12–14 kDa) for 3 days, then lyophilized (Hetodrywinner) and weighed (Finore et al., 2020). Phenol/sulfuric acid method (Dubois et al., 1956) was used to assay the total carbohydrate content of exopolymers. All experiments were carried out in duplicate.

### Ethanol Production and Quantification

The preculture of *P. thermantarcticus* strain M1 was incubated in anaerobic condition for determining the relation between microbial oxygen and the ethanol production capability. Therefore, after growth in strictly anaerobic medium, the broth was centrifuged at 10,000 rpm for 30 min at 4°C. The amount of ethanol produced by *P. thermantarcticus* strain M1 was determined from the cell-free supernatant using the Megazyme K-ETOH kit according to the manufacturer’s instructions.

### Accession Numbers of Nucleotide Sequence

The genome project of *P. thermoantarcticus* M1 has been deposited at DDBJ/EMBL/GenBank under the accession FOJS00000000.1. Accession number of the first version was FOJS00000000.1. The gene sequence of 16S rRNA was available in DDBJ/EMBL/GenBank with the accession number KF192950.

## Results

### General Features of the *Parageobacillus thermantarcticus* M1 Genome

The gene prediction and genome annotation of *P. thermantarcticus* M1 revealed 3,957 coding sequences and 86 RNA genes (Table 1). A total of 2,580 protein coding genes were assigned with putative functions, whereas 1,377 hypothetical proteins couldn’t be matched to any known protein. Coding sequences assigned to any subsystem made up twenty-ninth percent of the total coding sequences (Supplementary Materials 1, 2). Furthermore, the gene annotation analysis resulted in the presence of the complete glycolysis, gluconeogenesis, Entner-Doudoroff, pentose phosphate pathways, TCA cycle, and genes encoding enzymes needed for ethanol fermentation. Moreover, pathways for the biosynthesis of folate, thiamine, and riboflavin were found to be present in *P. thermantarcticus* genome.

**TABLE 1:** General features of *P. thermantarcticus* M1 draft genome.

Genome	*P. thermantarcticus* M1
Domain	Bacteria
Taxonomy	Bacteria; Terrabacteria group; Firmicutes; Bacilli; Bacillales; Bacillaceae; Parageobacillus; *Parageobacillusthermantarcticus*
Size	3,448,881 bp
G + C content	43.7%
Number of subsystems	318
Number of coding sequences	3,957
Proteins with functional assignments	2,580
N50 value	61,729
L50 value	15
CRISPR repeats	67
CRISPR spacer	64
CRISPR array	3
Number of RNAs CheckM Completeness CheckM Contamination	86 100 0.7

In order to enlighten the functional characteristics of *P. thermantarcticus* M1, the association of genes with the general cluster of orthologous group (COG) functional categories was analyzed, and 65% of the total protein-coding genes were found to be associated with cellular processing and signaling, information storage and processing, and metabolism related functional categories (Table 2).

**TABLE 2:** Number of genes associated with the general cluster of orthologous group (COG) functional categories.

COG code	Number of genes	Percentage	Description
**Cellular process and signaling**			
D	62	1.6004	Cell cycle control, cell division, chromosome partitioning
M	123	3.1750	Cell wall/membrane/envelope biogenesis
N	79	2.0390	Cell motility
O	117	3.0201	Post-translational modification, protein turnover, and chaperones
T	154	3.9752	Signal transduction mechanisms
U	58	1.4972	Intracellular trafficking, secretion, and vesicular transport
V	48	1.2390	Defense mechanisms
W	2	0.0516	Extracellular structures
Y	0	0	Nuclear structure
Z	0	0	Cytoskeleton
**Information storage and processing**			
A	0	0	RNA processing and modification
B	1	0.0258	Chromatin structure and dynamics
J	167	4.3108	Translation, ribosomal structure and biogenesis
K	231	5.9628	Transcription
L	237	6.1177	Replication, recombination and repair
**Metabolism**			
C	179	4.6205	Energy production and conversion
E	294	7.5891	Amino acid transport and metabolism
F	70	1.8069	Nucleotide transport and metabolism
G	215	5.5498	Carbohydrate transport and metabolism
H	135	3.4848	Coenzyme transport and metabolism
I	97	2.5039	Lipid transport and metabolism
P	187	4.8271	Inorganic ion transport and metabolism
Q	57	1.4713	Secondary metabolites biosynthesis, transport, and catabolism
**Poorly characterized**			
R	400	10.3252	General function prediction only
S	285	7.3567	Function unknown
-	1,169	17.4501	Not in COGs

### Phylogenomic Analysis

**FIGURE 1:**
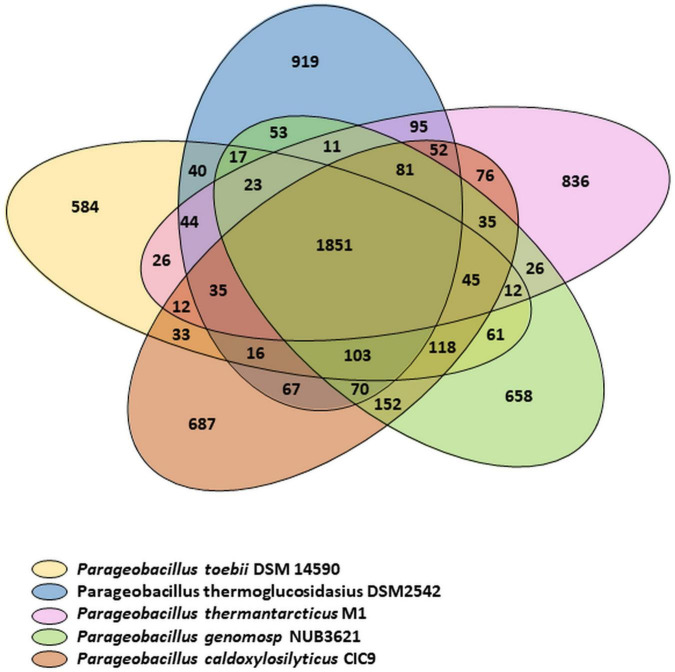
Venn diagram comparing the encoded proteins of *P. thermantarcticus* M1, *P. toebii* DSM 14590, *P. genomosp* NUB3621, *P. caldoxylosilyticus* CIC9, *P. thermoglucosidasius* DSM2542. The numbers of shared and unique proteins are shown.

The relationships between *P. thermantarcticus* M1 and four other species of *Parageobacillus* genus were analyzed by using putative function assignments of encoded proteins, which is called a phylogenomics approach (Figure 1; Miele et al., 2011). The pan-genome describes the entire set of genes known to exist in all members of a given genus, consisting of a core genome (a gene set commonly shared between all individuals of the species) and the dispensable or accessory genome (a gene set shared within only one or some strains) (Park et al., 2019). Family/gene numbers responsible for pan-genome, core-genome and variable genome were 6,838/18,697, 1,851/11,030, and 4,987/7,667, respectively. *P. thermantarcticus* M1 has 900 unique proteins assigned with putative functions that form 24.207% of the total CDS. *P. toebii* DSM 14590 has lowest variable CDS (1216 CDS that form 35.913% of total CDS) and strain specific CDS (592 CDS that form 17.484% of total CDS). Highest pan CDS (3951 CDS) and variable CDS (1717 CDS) as well as strain specific CDS (928 CDS) were assigned to *P. thermoglucosidasius* DSM2542. Numbers of genes for five different species are presented in Table 3. *P. thermantarcticus* M1 shared the highest number of gene family with *P. thermoglucosidasius* DSM 2542 (2192 CDS) and the lowest number of gene family with *P. toebii* DSM 14590 (2045 CDS).

**TABLE 3:** Pan/Core genome analysis of *Parageobacillus* genus.

Organism	Pan CDS	Core CDS	Var CDS	Strain specific CDS	Core CDS (%)	Var CDS (%)	Strain specific CDS (%)
*P. thermantarcticus M1*	3,718	2,155	1,563	900	57.961	42.039	24.207
*P. toebii DSM 14590*	3,386	2,170	1,216	592	64.087	35.913	17.484
*P. genomosp NUB3621*	3,731	2,212	1,519	670	59.287	40.713	17.958
*P. caldoxylosilyticus CIC9*	3,911	2,259	1,652	697	57.760	42.240	17.822
*P. thermoglucosidasius DSM2542*	3,951	2,234	1,717	928	56.593	43.457	23.488

### Stress Tolerance/Response

Cells are generally exposed to many environmental changes during industrial production processes and therefore, they develop stress response mechanisms to sense and combat the deleterious effects of various stresses. A total of 34 genes encoding proteins associated with stress response were found in *P. thermantarcticus* genome.

Under stress conditions, the expressions of stress responsive genes are re-organized. The transcription factors that interact with RNA polymerase are activated to coordinate the gene expression (Boor, 2006). In bacteria, sigma factors are required for transcription initiation. When a sigma factor associates with RNA polymerase, it directs promoter recognition. Since one subgroup of sigma factors comprises proteins that are activated in the presence of environmental stress (Balleza et al., 2008), the general stress regulons are controlled by the activity state of sigma factors. Therefore, the presence of a serine phosphatase RsbU (Pta.peg.737, Pta.peg.1300, Pta.peg.1512, Pta.peg.2721), which is a regulator of sigma subunit, in *P. thermantarcticus* genome was notable.

*P. thermantarcticus* showed two transporter genes encoding proteins that play roles in osmoregulation: glycerol uptake facilitator protein (Pta.peg.2109) and glycine betaine transporter OpuD (Pta.peg.3108) that is known from *Bacillus subtilis*. The OpuD system is a member of a small family of transport proteins involved in the accumulation of trimethylammonium compounds. It is a single component betaine glycine uptake system that actively participates in stress response of *B. subtilis* under high osmolarity environment (Kappes et al., 1996). The accumulation of glycine betaine at high concentrations was also reported in various bacterial species in response to osmotic stress (Kappes et al., 1996; Mandon et al., 2003; Wargo et al., 2008; Nau-Wagner et al., 2012; Ronzheimer et al., 2018).

Oxidative stress response is one of the key responses found in *P. thermantarcticus*. Genes encoding proteins involved in the regulation of oxidative stress response, such as nitrite-sensitive transcriptional repressor NsrR (Pta.peg.2043), peroxide stress regulator PerR, FUR family (Pta.peg.107), ferric uptake regulation protein FUR (Pta.peg.1861), alkyl hydroperoxide reductase subunit C-like protein (Pta.peg.957) were identified in the genome. Moreover, genes encoding a cytosolic enzyme glutathione peroxidase GPX (EC 1.11.1.9, Pta.peg.3266, and Pta.peg.3324) that catalyzes the reduction of hydrogen peroxide to water and oxygen and the reduction of peroxide radicals to alcohols and oxygen were detected. The protection of glutathione against oxidative stress was reported for several bacteria (Li et al., 2003; Richard and Love, 2006; Arenas et al., 2010). In addition, three superoxide dismutase (SOD) proteins, including superoxide dismutase [Fe] (EC 1.15.1.1, Pta.peg.1893), superoxide dismutase [Cu-Zn] precursor (EC 1.15.1.1, Pta.peg.428), and superoxide dismutase [Mn] (EC 1.15.1.1, Pta.peg.3553) were present in the genome. SOD proteins protect the cells from reactive oxygen species by catalyzing the dismutation of superoxide radicals (O_2_^–^) to either molecular oxygen (O_2_) or hydrogen peroxide (H_2_O_2_). Therefore, the presence of SOD proteins supported the existence of a possible defense mechanism against oxidative stress in *P. thermantarcticus*.

Other stress-related genes included a periplasmic stress related gene (intramembrane protease RasP/YluC, implicated in cell division based on FtsL cleavage, Pta.peg.3173), three bacterial hemoglobin genes (hemoglobin-like protein HbO, Pta.peg.2877, and di-guanylate cyclase/phosphodiesterase (GGDEF and EAL domains) with PAS/PAC sensors, Pta.peg.258 and Pta.peg.2815), two genes involved in carbon starvation (carbon starvation protein A, CstA, Pta.peg.2476, and carbon storage regulator, CsrA, Pta.peg.2671), and a *hfl* operon gene (RNA binding protein Hfq, Pta.peg.3104).

Finally, thermophilic microorganisms are able to not only tolerate high temperatures, but also exploit them to their advantage. Understanding high temperature tolerance of these microorganisms is essential for the analysis of thermophilic enzymes that can work at high temperatures. *P. thermantarcticus* M1 was found to have spermidine synthase (EC 2.5.1.16, Pta.peg.2044, and Pta.peg.2994) gene in its genome. The presence of the genes related to polyamine metabolism is common in the genome of thermophilic bacteria and considered to be associated with the thermophilicity of thermophiles (Goh et al., 2014, 2016). Caseinolytic protease (Clp) family proteins play important role in heat stress response. These proteins are high molecular weight chaperones that are part of the Heat Shock Protein 100 (HSP100) family (Erdayani et al., 2020). ClpB (Pta.peg.2905), ClpX (Pta.peg.3746), ClpC (Pta.peg.1716), ClpE (Pta.peg.1801) and ATP-dependent Clp protease proteolytic subunit (EC 3.4.21.92, Pta.peg.2605) were assigned in the genome of M1 strain. Moreover, the genome included genes encoding heat shock protein GrpE (Pta.peg.3602), chaperone proteins DnaK (Pta.peg.3601), DnaJ (Pta.peg.3600) and GroEL (Pta.peg.713), co-chaperone GroES (Pta.peg.712), and heat-inducible transcription repressor HrcA (Pta.peg.3603).

### Transport

Transport is a vitally important system for all organisms on the Earth. *P. thermantarcticus* M1 showed 272 open reading frames (ORFs) (6.9% of total genome) related with various transport systems. These transport systems of the bacterium take role in the accumulation of necessary nutrients, extrusion of undesirable by-products and maintenance of cytoplasmic content of ions, salts, etc. A total of 113 ORFs were assigned to transport associated subsystems, whereas 159 ORFs were not categorized under a subsystem despite their roles in transport. The products of these ORFs consist of transporters for sugars and carbohydrates, ions (F^–^,S^2–^, Mg^2+^, Zn^2+^, Mn^2+^, Fe^2+/3+^, Co^2+^, Ca^2+^, and Mo^2+^), amino acids (arginine, glutamine), and several further molecules (β-xyloside vitamin B12, spermidine/putrescine, glycerol-3-phosphate, polyamine, siderophore and subtilin) (Supplementary Materials 1, 2). Furthermore, the genome included genes responsible for quorum sensing, for instance those encoding ATP-binding cassette (ABC) transporters (Pta.peg. 211, 2887, 2888, 2889, 2890, 3462, 3463, 3464, 3465, 3466) and two-component sensor histidine kinase proteins (Pta.peg.516, 799, 850, 1450, 1634, 2229, 3454, 3504) for detection of the autoinducers.

### Uptake and Utilization of Carbon Sources

**FIGURE 2:**
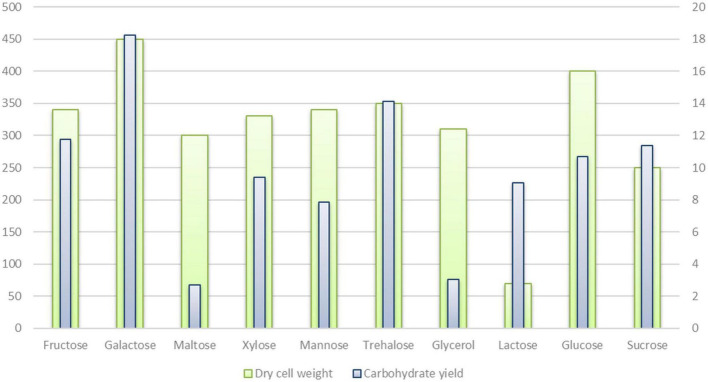
Carbohydrate utilization and exopolysaccaride production by *P. thermantarcticus* M1.

Genes coding the ABC transporters and transporters for carbon sources (ribose, xylose, arabinose, galactose, glycerol, maltose/maltodextrin, and lactose), and utilization systems for sucrose, fructose, raffinose, xylose, glycerol, lactose, galactose, ribose, and chitin were identified in *P. thermantarcticus* genome. Moreover, the phosphoenolpyruvate-dependent sugar phosphotransferase system (sugar PTS) that is a major carbohydrate active transport system were detected for trehalose (Pta.peg.2053), maltose (Pta.peg.2362), glucose (Pta.peg.2366), and fructose (Pta.peg.3377). In addition, genes encoding enzymes associated with glucose, mannose and trehalose metabolisms were detected (Supplementary Materials 1, 2). In order to validate sugar uptake and utilization, *P. thermantarcticus* were cultured in the presence of 10 different sugars as carbon sources. Growth profiles of cultures indicated that glucose, fructose, sucrose, galactose, lactose, maltose, xylose, glycerol, mannose, and trehalose were found to be utilized for growth and EPS production (Figure 2).

### Exopolysaccharide Biosynthesis

*P. thermantarcticus* was previously reported to synthesize two different EPSs: (i) EPS1 is a sulfate heteropolysaccharide composed of mannose and glucose (1.0:0.7 molar ratio), (ii) EPS2 is a sulfate homopolysaccharide composed of mannose as the major component (Manca et al., 1996). In the aforementioned study, the EPS fraction was obtained with all sugars tested, while a higher yield was obtained with mannose as the source of carbon and energy. Additionally, in this study, the EPS yields achieved from cultures grown in 10 different carbohydrates were compared (Figure 2 and Table 4). Although undetectable levels of EPSs were obtained in the presence of maltose and glycerol, the highest yields were reached by cultures grown on galactose (18.24 mg/l) and trehalose (14.12 mg/l).

**TABLE 4:** Carbohydrate uptake and utilization by *P. thermantarcticus* M1.

C-source	Transport system	Associated gene(s)
Fructose	PTS system	Pta.peg.3377
Galactose	ABC transporter	Pta.peg.687
Maltose	ABC transporter/PTS system	Pta.peg.416, 608, 790, 791, 793, 890, 891, 892, 3427, 3429, 3441, 3451/Pta.peg.2362
Xylose	ABC transporter	Pta.peg.687, 3450, 3452, 1449
Mannose	PTS system	Pta.peg.3394, 3395, 3396
Trehalose	PTS system	Pta.peg.2053
Glycerol	Glycerol uptake facilitator protein	Pta.peg.2109
Lactose	ABC transporter	Pta.peg.687
Glucose	PTS system	Pta.peg.2366
Sucrose	ABC transporter	Pta.peg.1261, 2473, 3022

### Ethanol Biosynthesis

*P. thermantarcticus* produced ethanol only in strictly anaerobic conditions, by using glucose as a carbon source, the maximum amount reached was 1.85 mg/l. The comparison of annotated *P. thermantarcticus* metabolic pathways to the glycolysis and oxidative decarboxylation of pyruvate deposited in KEGG resulted in the existence of the genes encoding enzymes required for ethanol synthesis from glucose. Firstly, glucose was converted to pyruvate by glycolysis pathway and then pyruvate is further used to form Acetyl-CoA through oxidative decarboxylation (Supplementary Material 2). Then, acetate was formed from Acetyl-CoA by acetyl-CoA synthetase (EC 6.2.1.1, Pta.peg.3869), and aldehyde dehydrogenase (EC 1.2.1.3, Pta.peg.671, Pta.peg.1191, Pta.peg.1463, Pta.peg.3297, Pta.peg.3404) catalyzed the acetaldehyde formation from acetate. Finally, the product, ethanol, was synthesized by alcohol dehydrogenase (EC 1.1.1.1, Pta.peg.820, Pta.peg.1274, Pta.peg.1800, Pta.peg.3497). The presence of genes encoding these enzymes showed the metabolic capacity of *P. thermantarcticus* to produce ethanol by utilizing glucose.

### Comparative *Parageobacillus* Genome Analysis

At time of writing, *Parageobacillus* includes 7 species and to date, a total of 4 WGS for the strains of the thermophilic species *P. caldoxylosilyticus* (Berendsen et al., 2016; Ching et al., 2022), *P. thermoglucosidasius* (Zhao et al., 2012; Brumm P. et al., 2015; Brumm P. J. et al., 2015; Chen et al., 2015; Sheng et al., 2016; Inoue et al., 2019), *P. toebii* (Berendsen et al., 2016; Sharma et al., 2019; Hatmaker et al., 2020), as well as *P. thermantarcticus* M1, were reported. Comparative analysis was performed for these 4 genomes on the basis of carbohydrate uptake/utilization and EPS biosynthesis mechanisms (Supplementary Material 3). All subsystems and proteins that are responsible for sugar uptake/utilization or EPS biosynthesis, but couldn’t be assigned to any subsystem, were also evaluated. Published literature and genome information of these 7 species indicated biological, biotechnological and industrial significance of *Parageobacillus* genus, as well as similarities and differences within the genus itself.

*Parageobacillus* species were able to utilize D-xylose. The *xyl*A gene encoding D-xylose isomerase (EC 5.3.1.5), and *xyl*B gene encoding D-xylulose kinase (EC 2.7.1.17) have been found in the genome of *P. caldoxylosilyticus*, *P. thermoglucosidasius*, *P. toebii* and *P. thermantarcticus.* The *xyl* gene cluster also comprised genes encoding the D-xylose transport function. Coding genes for D-xylose transportation, such as ATP-binding protein (*xyl*G), substrate-binding protein (*xyl*F) and transport permease protein (*xyl*H) were present in the genomes of all species. On the other hand, genes responsible for the utilization of L-arabinose were present only in the genome of *P. caldoxylosilyticus* and *P. thermoglucosidasius*. L-arabinose transporter genes *ara*G (ATP-binding protein) *ara*F (substrate-binding protein) *ara*H (permease protein), as well as *ara*A (arabinose isomerase) were present in these two genomes. However, *Parageobacillus* species were unable to ferment D-allose, and the utilization pathway of D-allose was completely absent in the genome of all species.

Chitin, a β-1,4-linked polymer of N-acetylglucosamine (GlcNAc), is the second most abundant polysaccharide on Earth, and mainly originates from the cuticle of arthropods and the cell wall of filamentous fungi (Zhao et al., 2010). The ability to metabolize chitin provides a competitive advantage to soil-dwelling microorganisms. Indeed, soils are generally carbon-rich and nitrogen-poor environments (Hodgson, 2000), and GlcNAc-containing polymers are a rich source for both essential nutrients. Subsystem of “Chitin and N-acetylglucosamine utilization” is present in the genome of four species.

D-Galacturonate and D-Glucuronate utilization and dTDP-rhamnose synthesis pathways were present in the genomes of *P. caldoxylosilyticus* and *P. thermatarcticus*. A total of 7 genes assigned to subsystem of “D-Galacturonate and D-Glucuronate utilization.” The gene cluster required for the degradation of D-glucuronate, including *uxa*C encoding uronate isomerase (EC 5.3.1.12), *uxu*B encoding D-mannonate oxidoreductase (EC 1.1.1.57) and *uxu*A encoding mannonate dehydratase (EC 4.2.1.8), was present in both *P. caldoxylosilyticus* and *P. thermatarcticus*. However, *uxa*B encoding altronate oxidoreductase (EC 1.1.1.58) was absent in all genomes and *uxa*A encoding altronate dehydratase (EC 4.2.1.7) was present only in the genome of *P. caldoxylosilyticus*. Pathway of dTDP-L-rhamnose synthesis from D-glucose-1P was complete in both *P. caldoxylosilyticus* and *P. thermatarcticus*. *Rml*A encoding glucose-1-phosphate thymidylyltransferase (EC 2.7.7.24), *rml*B encoding dTDP-glucose 4,6-dehydratase (EC 4.2.1.46), *rml*C encoding dTDP-4-dehydrorhamnose 3,5-epimerase (EC 5.1.3.13), and *rml*D encoding dTDP-4-dehydrorhamnose reductase (EC 1.1.1.133) gene cluster was annotated under “dTDP-rhamnose synthesis” subsystem.

There are three main routes for biosynthesis of microbial EPSs: (i) Wzx/Wzy-dependent pathway; (ii) ATP-binding cassette (ABC) transporter-dependent pathway, and (iii) synthase-dependent pathway (Wang et al., 2019). Fructan or glucan based homopolysaccharides are synthesized by sucrase enzymes (Rehm, 2009). These glucan-sucrases and fructan-sucrases act on sucrose, hydrolyze glycosidic bonds, and then polymer chain is formed by transglycosylation reactions. The absence of genes encoding these sucrose enzymes in these four *Parageobacillus* species showed the lack of glucan or fructan biosynthesis, indicating that the EPSs are synthesized intracellularly like most of the EPSs from extremophilic bacteria (Yildiz et al., 2015). The other two pathways for the synthesis of heteropolysaccharides and some homopolysaccharides are more complicated and requires the uptake of monosaccharides like glucose, xylose, fructose, mannose, or glycerol. Then, these monosaccharides are converted into nucleoside diphosphate sugars (NDP-sugars), and isoprenoid lipid–phosphate is formed in the cytoplasmic membrane assembly of NDP-sugars.

At this stage, repeating sugar monomer units are transferred sequentially from sugar nucleotides by glycosyltransferases, and then an acyl group is added for the modification, and lastly polymerization is completed. As a final point, the synthesized polysaccharide is secreted from the membrane of the cell into the extracellular environment (Sutherland, 1972). Genome analysis showed the existence of a number of genes that might have roles in four different stages of EPS biosynthesis mechanism (Table 5). Comparative analysis of the genomes revealed that most of the genes responsible for biosynthesis of NDP-sugars existed in all *Parageobacillus* species. Genes encoding enzymes/proteins in the biosynthesis of isoprenoid lipid–phosphate and several glycosyltransferases were found in all compared genomes. All species were found to have deacetylase enzymes; however, chitooligosaccharide deacetylase (EC 3.5.1.-) was annotated for all species except *P. thermantarcticus*. On the other hand, polysaccharide pyruvyl transferase was annotated for *P. thermantarcticus* and *P. caldoxylosilyticus* (Supplementary Material 3). Two main mechanisms exist for the polysaccharide transport system. In the first scenario, a polysaccharide can be exported by ABC transporters across the cytoplasmic membrane. The existence of the related genes in the genome of *P. thermantarcticus* M1 supported the presence of this mechanism for the EPS export (Table 5). Second scenario is the polymerization by a wzx/wzy-dependent pathway after isoprenoid-linked intermediates are flipped through the cytoplasmic membrane. Comparative genome analysis indicated that *Parageobacillus* species used first mechanism for the transport of polysaccharides (Supplementary Material 3).

**TABLE 5:** Predicted genes and proteins involved in EPS biosynthesis.

Steps of EPS biosynthesis/Metabolites	Essential gene(s)	Encoded protein(s)
**Biosynthesis of NDP-sugars**		
UDP-glucose	Pta.peg.1692/ Pta.peg.2221	UTP-glucose-1-phosphate uridylytransferase (EC 2.7.7.9)/Galactose-1-phosphate uridylyltransferase (EC 2.7.7.10)
UDP-galactose	Pta.peg.392, 393, 1131, 2222	UDP-glucose 4-epimerase (EC 5.1.3.2)
GDP-mannose	Pta.peg.3277	Mannose-1-phosphate guanylyltransferase (EC 2.7.7.13)
UDP- N-acetylglucosamine	Pta.peg.1569	N-acetylglucosamine-1-phosphate uridyltransferase (EC 2.7.7.23)/Glucosamine-1-phosphate N-acetyltransferase (EC 2.3.1.157)
UDP- N-acetylgalactosamine	Pta.peg.392, 393, 1131, 2222	UDP-N-acetylglucosamine 4-epimerase (EC 5.1.3.7)/UDP-glucose 4-epimerase (EC 5.1.3.2)
UDP- N-acetylmannosamine	Pta.peg.876	UDP-N-acetylglucosamine 2-epimerase (EC 5.1.3.14)
**Assembly on lipid-acceptor**		
Di-trans,octa-cis-undecaprenyl diphosphate	Pta.peg.3176	Undecaprenyl pyrophosphate synthetase (EC 2.5.1.31)
Di-trans,octa-cis-undecaprenyl phosphate	Pta.peg.408, 858	Undecaprenyl-diphosphatase (EC 3.6.1.27)
Glycosyltransferases		
	Pta.peg.1414, 1614, 1708, 2637, 2692, 3017, 3276	Glycosyltransferase (2.4.1.-)
	Pta.peg.1707	Glycosyltransferase family 1 (2.4.1.-)
	Pta.peg.1694	Glycosyltransferase family 2 (2.4.1.-)
	Pta.peg.1360	Glycosyltransferase family 8 (2.4.1.-)
	Pta.peg.864	Poly (glycerol-phosphate) α-glucosyltransferase (EC 2.4.1.52)
	Pta.peg.3275	Mannosyltransferase (2.4.1.-)
	Pta.peg.1693	Undecaprenyl-phosphate galactosephosphotransferase (EC 2.7.8.6)
	Pta.peg.628	UDP-N-acetylglucosamine–N-acetylmuramyl-(pentapeptide) pyrophosphoryl-undecaprenol N-acetylglucosamine transferase (EC 2.4.1.227)
	Pta.peg.2689	Undecaprenyl-phosphate α-N-acetylglucosaminyl 1-phosphate transferase (EC 2.7.8.33)
	Pta.peg.2256	UDP-N-acetylglucosamine:L-malate glycosyltransferase
	Pta.peg.2995, 3868	Multimodular transpeptidase-transglycosylase (EC 2.4.1.129) (EC 3.4.-.-)
	Pta.peg.865	N-acetylmannosaminyltransferase (EC 2.4.1.187)
**Modification**		
	Pta.peg.3945	Glycogen phosphorylase (EC 2.4.1.1)
	Pta.peg.867	Polysaccharide pyruvyl transferase
	Pta.peg.743,1786	Polysaccharide deacetylase
**Transport of EPS**		
	Pta.peg.2269, 2612, 3543, 3658, 3748	TPR repeat protein
	Pta.peg.3037, 3865	GAF domain/HD domain protein/GGDEF domain
	Pta.peg.78, 116, 401, 788, 891, 1167, 1387, 2833	ABC transporter, ATP binding protein
	Pta.peg.416, 687, 793, 890, 1257	ABC transporter, sugar binding protein
	Pta.peg.609, 791, 892, 1258, 1259, 3451	ABC transporter, permease protein
	Pta.peg.91, 92, 254, 784, 851, 986, 1022, 1337, 1521, 1632	Lipoprotein

**FIGURE 3:**
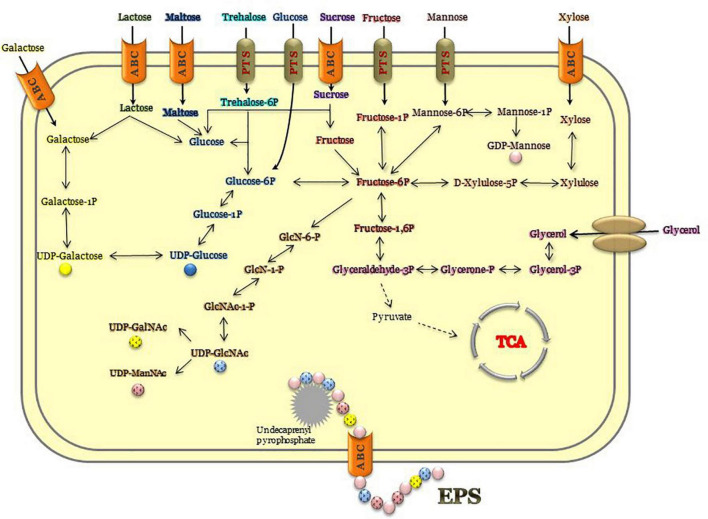
Proposed C-source utilization and EPS biosynthesis mechanism of *P. thermantarcticus* M1.

Essential genes associated with EPS biosynthesis were detected by genome annotation and together with experimental evidences, a hypothetical mechanism for EPS biosynthesis was generated (Figure 3). In this mechanism, the pathways for sugar uptake as well as the biosynthesis of NDP-sugars were shown. However, the annotated glycosyltransferases (Table 5) should be characterized more specifically such that their role in the whole process can be identified more precisely.

### Features of Industrial and Biotechnological Interest

**FIGURE 4:**
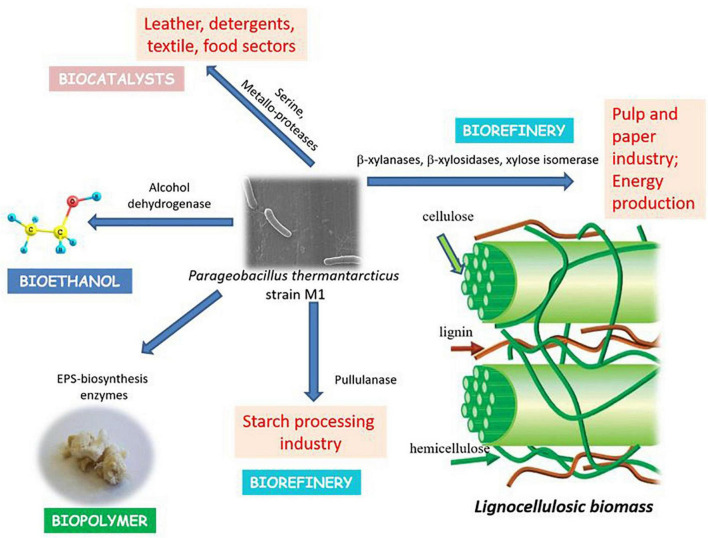
Industrial and biotechnological features of *P. thermantarcticus* M1.

Thermophilic microorganisms are of the utmost importance considering the production of thermostable enzymes (Finore et al., 2019). WGS of *P. thermantarcticus* M1 revealed the presence of many industrially important enzymes, including lipases, esterases, proteases, xylanases, β-xylosidases, and xylose isomerases (Figure 4 and Table 6).

**TABLE 6:** Industrially important enzymes predicted by genome analysis of *P. thermantarcticus* M1.

Enzyme name	EC number	Associated gene(s)
Lipase	EC 3.1.1.-	Pta.peg.2190, 2553, 2708, 3304, 3412, 3912
Esterase	EC 3.1.1.-/ EC 3.1.4.-	Pta.peg.258, 379, 606, 621, 916, 1406, 1612, 1849, 2472, 2520, 2594, 2596, 2792, 2815, 2847, 2848, 2937, 3058, 3188, 3350, 3389, 3757
β-Xylanase	EC 3.2.1.8	Pta.peg.3417, 3446
β-Xylosidase	EC 3.2.1.37	Pta.peg.3425, 3447
Xylose isomerase	EC 5.3.1.5	Pta.peg.3410
Pullulanase	EC 3.2.1.41	Pta.peg.3892
Neopullulanase	EC 3.2.1.135	Pta.peg.1260
Serine protease	EC 3.4.21.-	Pta.peg.161, 2372, 3049
Metalloprotease	EC 3.4.24.-	Pta.peg.2997
Nitrilase	EC 3.5.5.4	Pta.peg.3281
Amidase	EC 3.5.1.-	Pta.peg.219, 595, 684, 874, 1193, 1782, 2131, 3134, 3357, 3515, 3517, 3593
Dipeptidase	EC 3.4.13.19	Pta.peg.3127, 3841, 3896
Aldo-Keto reductase	EC 1.1.1.-	Pta.peg.164, 1080
N-acetylglucosamine-6-phosphate deacetylase	EC 3.5.1.25	Pta.peg.1904
Glucosamine-6-phosphate deaminase	EC 3.5.99.6	Pta.peg.1905

β-xylanases and β-xylosidases are required for complete hydrolysis and assimilation of β-1,4-xylan, which is the most important hemicellulose component of lignocellulosic biomass. Xylanases, which have been mostly reported in bacteria and fungi, play a crucial role in pulp and paper industry by hydrolyzing xylan, and consequently releasing lignin from paper pulp, and reducing the use of chlorine as the bleaching agent (Lama et al., 2004). In industrial processes, xylanases obtained from thermophilic organisms are preferred and *Geobacillus* genus come to prominence due its ability to utilize various carbon sources. Bibra et al. (2018) reported the production of a highly thermostable xylanase from *Geobacillus sp.* strain DUSELR13, which had the potential in bioethanol production processes. Genome annotation analysis showed the presence of xylanolytic enzymes, β-xylanase and β-xylosidase, in *P. thermantarcticus* M1 genome. Moreover, the production of both enzymes by *P. thermantarcticus* M1 was previously reported and the characterization of these enzymes showed their potential use in various biotechnological applications (Lama et al., 2004). In addition, the presence of the gene encoding alcohol dehydrogenase (EC 1.1.1.1, Pta.peg.820, Pta.peg.1274, Pta.peg.1800, Pta.peg.3497), that converts acetaldehyde to ethanol, endowed the strain with application in bioethanol production from lignocellulosic biomass.

Hemicelluloses can be used as a sole carbon source for growth of various microorganisms. Initially, hemicelluloses are degraded to D-xylose by exoenzymes and then D-xylose is transported into the cell. Xylose isomerase is required for the isomerization of D-xylose to D-xylulose. Lama et al. (2001) reported the production of a thermostable xylose isomerase by *P. thermantarcticus*. Genome annotation analysis showed the presence of the gene encoding xylulose kinase (EC 2.7.1.17, Pta.peg.3409) that catalyzes the phosphorylation reaction of D-xylulose to D-xylulose 5-phosphate, and the gene encoding ribulose-phosphate 3-epimerase (EC 5.1.3.1, Pta.peg.3249) that is required for the conversion of D-xylulose 5-phosphate to D-ribulose 5-phosphate, which enters the pentose phosphate pathway in *P. thermantarcticus* M1 genome.

*P. thermantarcticus* M1 genome carried a gene encoding pullulanase, a pullulan degrading enzyme. Pullulanase is known to play a crucial role in starch processing, leading to its widespread use in various applications in food, pharmaceutical, material, and bioenergy industries (Pang et al., 2020). Moreover, the analysis indicated the presence of a gene encoding neopullulanase, a type I pullulan hydrolase (Kahar et al., 2022). These pullulan degrading enzymes can hydrolyze the glycosidic bonds of pullulan and starch, and forms reducing sugars. Therefore, the presence of pullulan degrading enzymes in *P. thermantarcticus* M1 might lead to its potential application in bioethanol production from starchy biomass.

*P. thermantarcticus* M1 genome also carried multiple genes encoding proteases, specifically serine and metalloproteases, that constitute one of the most important group of industrial enzymes. The production of protease enzyme from *P. thermantarcticus* M1 was previously reported and its characterization revealed its potential use in biotechnological applications (Dipasquale et al., 2008). However, future studies are required for experimental validation of the other enzymes presented in Table 6.

## Discussion

The whole genome sequencing of *P. thermantarcticus* M1 was performed to elucidate the genetic and metabolic interrelations of this thermophilic bacteria and to enhance our understanding on its potential in industrial applications. *P. thermantarcticus* M1 was able to produce highly thermostable enzymes and degrade hemicellulose. In particular, it possessed the genes encoding both exo- and endo-xylanolytic enzymes, that are required for the decomposition of lignocellulosic biomass. Together with the previous experimental data, the whole genome analysis made this microorganism a promising cell factory for the development of bioprocesses that convert lignocellulosic waste into biofuels and value-added bioproducts according to biorefinery approach (Finore et al., 2019).

*P. thermantarcticus* M1 genome annotation showed the presence of genes encoding protease and glucose (xylose) isomerase, underlying the great potential of this Antarctic bacteria in industrially important biotechnological applications. Furthermore, the identification of genes encoding many further valuable catalysts, such as lipase, esterase, pullulanase, aldo-keto reductase, and enzymes that plays roles in chitin utilization could contribute to the development of important industrial processes. In recent years, microbial esterases attracted much attention for their potential use in the degradation of plastics and microplastics due to their presence in all kind of environments (Mohanan et al., 2020). Today, the existence of microplastics reach up to the food chain and affects human health (Othman et al., 2021). Microplastic contamination is commonly caused by anthropogenic activities, such that plastics are used daily by humans and come from a wide range of sources from industry sectors to domestic activities. Increasing the yields of enzymatic degradation plays great importance in microplastics degradation. Moreover, the identification of novel enzymes through proteomic approach can be an outstanding technique to solve this environmental problem.

The aldo-ketoreductase superfamily comprises enzymes involved in detoxification, and act on substrates such as environmental pollutants, a large number of pharmaceuticals, drugs, and xenobiotics (Barski et al., 2008). Therefore, the presence of the gene encoding an aldo-keto reductase is of fundamental importance in the discovery of new sources for biodegradation of pollutants and drugs from microbial origin. Moreover, the genome analysis indicated the presence of “Chitin and N-acetylglucosamine Utilization” subsystem. The ability to metabolize chitin, which is the second most abundant polysaccharide on Earth, provides a competitive advantage to soil-dwelling microorganisms (Hodgson, 2000).

The identification of the gene encoding alcohol dehydrogenase that is required for ethanol production, could make this thermophile an ideal candidate for bioethanol production. *P. thermantarcticus* M1 genome was also found to encode for xylanolytic enzymes, and pullulanase involved in lignocellulose and starch degradation, respectively. These features unveiled the potential of this strain as a cell factory for bioethanol production from lignocellulosic biomass and/or starchy waste products.

The genes involved in quorum sensing mechanism, were also detected by genome annotation. At the time of writing, quorum sensing mechanism of *P. thermantarcticus* M1 has not yet studied and therefore, our findings open new insights for the exploitation of cell-cell communication, and communications between cells of different species, that is important for the coordination of community behavior and for the inhibition of competing species.

Apart from the microbial capability, many cellular mechanisms that allow *P. thermantarcticus* M1 to survive under stressful conditions, were elucidated by genome annotation. Cells encounter with stressful conditions during industrial production processes, and develop stress response mechanisms to combat the deleterious effects of various stresses. Genome annotation analysis revealed the genes specifically associated with osmotic and oxidative stresses *P. thermantarcticus* M1 genome.

Another interesting aspect addressed was the ability of *P. thermantarcticus* M1 to produce EPS from a variety of sugars. Our results indicated the developed versatility of the bacterium that enables it to utilize different types of carbohydrates as carbon and energy source for both growth and EPS production. Combined with its strong lignocellulolytic activity, the wide spectrum of carbon source utilization made *P. thermantarcticus* M1 to recruit the use of mixed sugars as substrate for EPS production through thermophilic bioprocessing.

Essential genes associated with EPS biosynthesis were detected by genome annotation, and together with the experimental evidences showed that the Antarctic microorganism transported the required EPS monomers across the cytoplasmic membrane by ABC transporters and produce EPS outside the cells. In microbial EPS production, the knowledge of mechanism is needed for process optimization, and improvement of the product quality. Since the genetic information of *P. thermantarcticus*, such as genome position, coding region, gene product function, and Enzyme Commission (EC) numbers, was identified by genome annotation, this study would play a significant role in the reconstruction of a metabolic model of this extremophilic microorganism. The elucidation of the pathways and molecular mechanisms leading to EPS production would help the development of metabolic engineering strategies for *P. thermantarcticus* in order to optimize medium, to enhance biopolymer production yields, to lower the production costs, and to improve the quality and efficiency of the process for industrial-scale applications.

This study clearly established the biotechnological and industrial potential of *P. thermantarcticus* M1. Although further experimental evidences are required to elucidate the underlying molecular mechanisms and to clarify the metabolism of this thermophilic bacterium, the genome annotation information would be crucial for further *in silico* and *in vivo* studies. Moreover, this understanding would help to design engineering strategies that lead to the optimized production processes resulted in improved properties of value-added industrial products, especially EPSs and biofuels.

## Data Availability Statement

The datasets presented in this study can be found in online repositories. The names of the repository/repositories and accession number(s) can be found below: https://www.ncbi.nlm.nih.gov/genbank/, (FOJS00000000.1, NZ_CP012712.1, RCWX00000000.1, AMRO00000000.1, and AOTZ00000000.1).

## Author Contributions

IF, AP, ET, CK, and SY conceived the study. CK and SY carried out the genome analysis. IF and AP designed the experiments. IF, IR, and LLe carried out the experiments. LLa and BN analyzed the experimental data. CK, SY, ET, IF, and AP wrote the manuscript. All authors contributed to the article and approved the submitted version.

## Conflict of Interest

The authors declare that the research was conducted in the absence of any commercial or financial relationships that could be construed as a potential conflict of interest.

## Publisher’s Note

All claims expressed in this article are solely those of the authors and do not necessarily represent those of their affiliated organizations, or those of the publisher, the editors and the reviewers. Any product that may be evaluated in this article, or claim that may be made by its manufacturer, is not guaranteed or endorsed by the publisher.

## Footnotes

1
https://img.jgi.doe.gov/cgi-bin/m/main.cgi

2
http://rast.nmpdr.org/

3
https://www.patricbrc.org/

4
http://blast.ncbi.nlm.nih.gov/Blast.cgi

5
http://www.uniprot.org/

6
http://www.ncbi.nlm.nih.gov/

7
http://www.genome.jp/kegg/

8
http://www.expasy.org/

9
https://www.genoscope.cns.fr/agc/microscope/home/index.php
