# Supplementary material for: Genomic Analysis Provides New Insights Into Biotechnological and Industrial Potential of Parageobacillus thermantarcticus M1
Source: Front Microbiol. 2022 Jun 9;13:923038. doi: 10.3389/fmicb.2022.923038 (PMC9218356; doi:10.3389/fmicb.2022.923038)
Supplement: Supplementary file 3 [file Table_3.docx]

| **Supplementary Table 3.** Comparison of *Parageobacillus* genomes according to genes associated with carbohydrate utilization and exopolysaccharide production | | | | | | | | | | | | |
| --- | --- | --- | --- | --- | --- | --- | --- | --- | --- | --- | --- | --- |
|  |  |  |  |  |  |  |  |  |  |  |  |  |
|  |  |  |  |  |  |  |  |  |  |  |  |  |
| **M1** | ***Parageobacillus thermantarcticus* M1** |  |  |  |  |  |  |  |  |  |  |  |
| **CIC9** | ***Parageobacillus caldoxylosilyticus* CIC9** |  |  |  |  |  |  |  |  |  |  |  |
| **DSM 14590** | ***Parageobacillus toebii* DSM 14590** |  |  |  |  |  |  |  |  |  |  |  |
| **DSM 2542** | ***Parageobacillus thermoglucosidasius* DSM 2542** |  |  |  |  |  |  |  |  |  |  |  |
|  |  |  |  |  |  |  |  |  |  |  |  |  |
| ■ present in the genome □ not present in the genome   \| **Subsystem** \| **Function** \| **M1** \| **CIC9** \| **DSM 14590** \| **DSM 2542** \| \| --- \| --- \| --- \| --- \| --- \| --- \| \| Beta-Glucoside Metabolism \| Outer surface protein of unknown function, cellobiose operon \| 0 \|  \|  \|  \| \| Beta-Glucoside Metabolism \| Transcriptional antiterminator of lichenan operon, BglG family \| 0 \|  \|  \|  \| \| Beta-Glucoside Metabolism \| Cellobiose phosphotransferase system YdjC-like protein \| 0 \|  \|  \|  \| \| Biotin synthesis cluster \| Competence protein F homolog, phosphoribosyltransferase domain \|  \|  \|  \|  \| \| Biotin synthesis cluster \| Substrate-specific component BioY of biotin ECF transporter \|  \|  \|  \|  \| \| Biotin synthesis cluster \| Biotin operon repressor \|  \|  \|  \|  \| \| Biotin synthesis cluster \| Adenosylmethionine-8-amino-7-oxononanoate aminotransferase (EC 2.6.1.62) \|  \|  \|  \|  \| \| Biotin synthesis cluster \| 8-amino-7-oxononanoate synthase (EC 2.3.1.47) \|  \|  \|  \|  \| \| Biotin synthesis cluster \| Dethiobiotin synthetase (EC 6.3.3.3) \|  \|  \|  \|  \| \| Biotin synthesis cluster \| Biotin synthase (EC 2.8.1.6) \|  \|  \|  \|  \| \| Chitin and N-acetylglucosamine utilization \| Glucosamine-6-phosphate deaminase (EC 3.5.99.6) \|  \|  \|  \|  \| \| Chitin and N-acetylglucosamine utilization \| N-acetylglucosamine-6-phosphate deacetylase (EC 3.5.1.25) \|  \|  \|  \|  \| \| Chitin and N-acetylglucosamine utilization \| Predicted transcriptional regulator of N-Acetylglucosamine utilization, GntR family \|  \|  \|  \|  \| \| Chitin and N-acetylglucosamine utilization \| N-Acetyl-D-glucosamine ABC transport system, permease protein 1 \|  \|  \|  \|  \| \| D-galactarate, D-glucarate and D-glycerate catabolism - gjo \| Sugar diacid utilization regulator SdaR \|  \|  \|  \|  \| \| D-Galacturonate and D-Glucuronate Utilization \| Transcriptional regulator KdgR, KDG operon repressor \|  \|  \|  \|  \| \| D-Galacturonate and D-Glucuronate Utilization \| Mannonate dehydratase (EC 4.2.1.8) \|  \|  \|  \|  \| \| D-Galacturonate and D-Glucuronate Utilization \| D-mannonate oxidoreductase (EC 1.1.1.57) \|  \|  \|  \|  \| \| D-Galacturonate and D-Glucuronate Utilization \| Uronate isomerase (EC 5.3.1.12) \|  \|  \|  \|  \| \| D-Galacturonate and D-Glucuronate Utilization \| Alpha-glucosidase (EC 3.2.1.20) \|  \|  \|  \|  \| \| D-Galacturonate and D-Glucuronate Utilization \| 2-dehydro-3-deoxyphosphogluconate aldolase (EC 4.1.2.14) \|  \|  \|  \|  \| \| D-Galacturonate and D-Glucuronate Utilization \| 2-keto-3-deoxygluconate permease (KDG permease) \|  \|  \|  \|  \| \| D-gluconate and ketogluconates metabolism \| Gluconate transporter family protein \|  \|  \|  \|  \| \| D-gluconate and ketogluconates metabolism \| Gluconokinase (EC 2.7.1.12) \|  \|  \|  \|  \| \| D-ribose utilization \| Ribose ABC transport system, ATP-binding protein RbsA (TC 3.A.1.2.1) \|  \|  \|  \|  \| \| D-ribose utilization \| Ribose operon repressor \|  \|  \|  \|  \| \| D-ribose utilization \| Ribose ABC transport system, permease protein RbsC (TC 3.A.1.2.1) \|  \|  \|  \|  \| \| D-ribose utilization \| Ribose ABC transport system, periplasmic ribose-binding protein RbsB (TC 3.A.1.2.1) \|  \|  \|  \|  \| \| dTDP-rhamnose synthesis \| dTDP-4-dehydrorhamnose reductase (EC 1.1.1.133) \|  \|  \|  \|  \| \| dTDP-rhamnose synthesis \| dTDP-glucose 4,6-dehydratase (EC 4.2.1.46) \|  \|  \|  \|  \| \| dTDP-rhamnose synthesis \| dTDP-4-dehydrorhamnose 3,5-epimerase (EC 5.1.3.13) \|  \|  \|  \|  \| \| dTDP-rhamnose synthesis \| Glucose-1-phosphate thymidylyltransferase (EC 2.7.7.24) \|  \|  \|  \|  \| \| Exopolysaccharide Biosynthesis \| Manganese-dependent protein-tyrosine phosphatase (EC 3.1.3.48) \|  \|  \|  \|  \| \| Exopolysaccharide Biosynthesis \| Tyrosine-protein kinase transmembrane modulator EpsC \|  \|  \|  \|  \| \| Exopolysaccharide Biosynthesis \| Tyrosine-protein kinase EpsD (EC 2.7.10.2) \|  \|  \|  \|  \| \| Exopolysaccharide Biosynthesis \| Undecaprenyl-phosphate galactosephosphotransferase (EC 2.7.8.6) \|  \|  \|  \|  \| \| Fructooligosaccharides(FOS) and Raffinose Utilization \| Multiple sugar ABC transporter, substrate-binding protein \|  \|  \|  \|  \| \| Fructooligosaccharides(FOS) and Raffinose Utilization \| Multiple sugar ABC transporter, membrane-spanning permease protein MsmG \|  \|  \|  \|  \| \| Fructooligosaccharides(FOS) and Raffinose Utilization \| Multiple sugar ABC transporter, membrane-spanning permease protein MsmF \|  \|  \|  \|  \| \| Fructose utilization \| Phosphoenolpyruvate-protein phosphotransferase of PTS system (EC 2.7.3.9) \|  \|  \|  \|  \| \| Fructose utilization \| Transcriptional repressor of the fructose operon, DeoR family \|  \|  \|  \|  \| \| Fructose utilization \| 1-phosphofructokinase (EC 2.7.1.56) \|  \|  \|  \|  \| \| Fructose utilization \| PTS system, fructose-specific IIB component (EC 2.7.1.69) \|  \|  \|  \|  \| \| Fructose utilization \| PTS system, fructose-specific IIC component (EC 2.7.1.69) \|  \|  \|  \|  \| \| Glycerate metabolism \| Hydroxypyruvate isomerase (EC 5.3.1.22) \|  \|  \|  \|  \| \| Glycerate metabolism \| 2-hydroxy-3-oxopropionate reductase (EC 1.1.1.60) \|  \|  \|  \|  \| \| Glycerate metabolism \| D-glycerate transporter (predicted) \|  \|  \|  \|  \| \| Glycerol and Glycerol-3-phosphate Uptake and Utilization \| Glycerol-3-phosphate ABC transporter, permease protein UgpE (TC 3.A.1.1.3) \|  \|  \|  \|  \| \| Glycerol and Glycerol-3-phosphate Uptake and Utilization \| Glycerophosphoryl diester phosphodiesterase, periplasmic (EC 3.1.4.46) \|  \|  \|  \|  \| \| Glycerol and Glycerol-3-phosphate Uptake and Utilization \| Glycerol-3-phosphate dehydrogenase [NAD(P)+] (EC 1.1.1.94) \|  \|  \|  \|  \| \| Glycerol and Glycerol-3-phosphate Uptake and Utilization \| Glycerol-3-phosphate ABC transporter, permease protein UgpA (TC 3.A.1.1.3) \|  \|  \|  \|  \| \| Glycerol and Glycerol-3-phosphate Uptake and Utilization \| Glycerol kinase (EC 2.7.1.30) \|  \|  \|  \|  \| \| Glycerol and Glycerol-3-phosphate Uptake and Utilization \| Glycerol-3-phosphate responsive antiterminator (mRNA-binding) \|  \|  \|  \|  \| \| Glycerol and Glycerol-3-phosphate Uptake and Utilization \| Glycerophosphoryl diester phosphodiesterase (EC 3.1.4.46) \|  \|  \|  \|  \| \| Glycerol and Glycerol-3-phosphate Uptake and Utilization \| Glycerol-3-phosphate ABC transporter, ATP-binding protein UgpC (TC 3.A.1.1.3) \|  \|  \|  \|  \| \| Glycerol and Glycerol-3-phosphate Uptake and Utilization \| GlpG protein (membrane protein of glp regulon) \|  \|  \|  \|  \| \| Glycogen metabolism \| Glucose-1-phosphate adenylyltransferase (EC 2.7.7.27) \|  \|  \|  \|  \| \| Glycogen metabolism \| 1,4-alpha-glucan (glycogen) branching enzyme, GH-13-type (EC 2.4.1.18) \|  \|  \|  \|  \| \| Glycogen metabolism \| Glycogen biosynthesis protein GlgD, glucose-1-phosphate adenylyltransferase family \|  \|  \|  \|  \| \| Glycogen metabolism \| Glycogen phosphorylase (EC 2.4.1.1) \|  \|  \|  \|  \| \| Glycogen metabolism \| Glycogen synthase, ADP-glucose transglucosylase (EC 2.4.1.21) \|  \|  \|  \|  \| \| Lactose and Galactose Uptake and Utilization \| Galactose-1-phosphate uridylyltransferase (EC 2.7.7.10) \|  \|  \|  \|  \| \| Lactose and Galactose Uptake and Utilization \| Galactokinase (EC 2.7.1.6) \|  \|  \|  \|  \| \| Lactose and Galactose Uptake and Utilization \| Aldose 1-epimerase (EC 5.1.3.3) \|  \|  \|  \|  \| \| L-Arabinose utilization \| L-ribulose-5-phosphate 4-epimerase (EC 5.1.3.4) \|  \|  \|  \|  \| \| L-Arabinose utilization \| Ribulokinase (EC 2.7.1.16) \|  \|  \|  \|  \| \| L-Arabinose utilization \| L-arabinose isomerase (EC 5.3.1.4) \|  \|  \|  \|  \| \| L-Arabinose utilization \| Transcriptional repressor of arabinoside utilization operon, GntR family \|  \|  \|  \|  \| \| Mannose Metabolism \| Phosphomannomutase (EC 5.4.2.8) \|  \|  \|  \|  \| \| Mannose Metabolism \| Mannose-6-phosphate isomerase (EC 5.3.1.8) \|  \|  \|  \|  \| \| Mannose Metabolism \| PTS system, mannose-specific IIB component (EC 2.7.1.69) \|  \|  \|  \|  \| \| Mannose Metabolism \| PTS system, mannose-specific IIC component (EC 2.7.1.69) \|  \|  \|  \|  \| \| Mannose Metabolism \| PTS system, mannose-specific IIA component (EC 2.7.1.69) \|  \|  \|  \|  \| \| Mannose Metabolism \| Beta-mannosidase (EC 3.2.1.25) \|  \|  \|  \|  \| \| Mannose Metabolism \| Mannose-1-phosphate guanylyltransferase (GDP) (EC 2.7.7.22) \|  \|  \|  \|  \| \| N-linked Glycosylation in Bacteria \| UDP-N-acetylglucosamine 4,6-dehydratase (EC 4.2.1.-) \|  \|  \|  \|  \| \| N-linked Glycosylation in Bacteria \| Lipid carrier : UDP-N-acetylgalactosaminyltransferase (EC 2.4.1.-) \|  \|  \|  \|  \| \| N-linked Glycosylation in Bacteria \| 4-keto-6-deoxy-N-Acetyl-D-hexosaminyl-(Lipid carrier) aminotransferase \|  \|  \|  \|  \| \| None \| ABC transporter, ATP-binding protein \|  \|  \|  \|  \| \| None \| Phosphoglucomutase (EC 5.4.2.2) \|  \|  \|  \|  \| \| None \| Undecaprenyl-diphosphatase (EC 3.6.1.27) \|  \|  \|  \|  \| \| None \| UDP-glucose 6-dehydrogenase (EC 1.1.1.22) \|  \|  \|  \|  \| \| None \| ABC transporter, substrate-binding protein (cluster 1, maltose/g3p/polyamine/iron) \|  \|  \|  \|  \| \| None \| Glucose-6-phosphate isomerase (EC 5.3.1.9) \|  \|  \|  \|  \| \| None \| Maltose/maltodextrin transport ATP-binding protein MalK (EC 3.6.3.19) \|  \|  \|  \|  \| \| None \| putative sugar ABC transporter, permease protein \|  \|  \|  \|  \| \| None \| Chitooligosaccharide deacetylase (EC 3.5.1.-) \|  \|  \|  \|  \| \| None \| UDP-N-acetylglucosamine--N-acetylmuramyl-(pentapeptide) pyrophosphoryl-undecaprenol N-acetylglucosamine transferase (EC 2.4.1.227) \|  \|  \|  \|  \| \| None \| UDP-N-acetylenolpyruvoylglucosamine reductase (EC 1.3.1.98) \|  \|  \|  \|  \| \| None \| ABC transporter, substrate-binding protein (cluster 2, ribose/xylose/arabinose/galactose) \|  \|  \|  \|  \| \| None \| Polysaccharide deacetylase \|  \|  \|  \|  \| \| None \| ABC transporter, permease protein 2 (cluster 1, maltose/g3p/polyamine/iron) \|  \|  \|  \|  \| \| None \| Phosphatidylethanolamine N-methyltransferase (EC 2.1.1.17) \|  \|  \|  \|  \| \| None \| Membrane protein of EXOQ family, involved in exopolysaccharide production \|  \|  \|  \|  \| \| None \| Poly(glycerol-phosphate) alpha-glucosyltransferase (EC 2.4.1.52) \|  \|  \|  \|  \| \| None \| N-acetylmannosaminyltransferase (EC 2.4.1.187) \|  \|  \|  \|  \| \| None \| UDP-N-acetyl-D-mannosamine dehydrogenase (EC 1.1.1.336) \|  \|  \|  \|  \| \| None \| Polysaccharide pyruvyl transferase \|  \|  \|  \|  \| \| None \| Lipopolysaccharide biosynthesis \|  \|  \|  \|  \| \| None \| ABC transporter, ATP-binding protein (cluster 1, maltose/g3p/polyamine/iron) \|  \|  \|  \|  \| \| None \| ABC transporter, permease protein 1 (cluster 1, maltose/g3p/polyamine/iron) \|  \|  \|  \|  \| \| None \| Glycerol-3-phosphate ABC transporter, substrate-binding protein UgpB \|  \|  \|  \|  \| \| None \| SN-glycerol-3-phosphate transport ATP-binding protein UgpC (TC 3.A.1.1.3) \|  \|  \|  \|  \| \| None \| sugar and carbohydrate transporters \|  \|  \|  \|  \| \| None \| Sensory box/GGDEF family protein \|  \|  \|  \|  \| \| None \| Maltose operon transcriptional repressor MalR, LacI family \|  \|  \|  \|  \| \| None \| 6-phospho-3-hexuloisomerase (EC 5.3.1.27) \|  \|  \|  \|  \| \| None \| D-arabino-3-hexulose 6-phosphate formaldehyde-lyase (EC 4.1.2.43) \|  \|  \|  \|  \| \| None \| Glycosyl transferase, family 8 \|  \|  \|  \|  \| \| None \| Glycosyltransferase \|  \|  \|  \|  \| \| None \| Sorbitol dehydrogenase (EC 1.1.1.14) \|  \|  \|  \|  \| \| None \| 6-phospho-beta-glucosidase (EC 3.2.1.86) \|  \|  \|  \|  \| \| None \| Predicted beta-glucoside-regulated ABC transport system, sugar binding component, COG1653 \|  \|  \|  \|  \| \| None \| Predicted beta-glucoside-regulated ABC transport system, permease component 1, COG1175 \|  \|  \|  \|  \| \| None \| Predicted beta-glucoside-regulated ABC transport system, permease component 2, COG0395 \|  \|  \|  \|  \| \| None \| Uncharacterized membrane protein YkoS \|  \|  \|  \|  \| \| None \| Uncharacterized glycosyltransferase YkoT \|  \|  \|  \|  \| \| None \| UTP--glucose-1-phosphate uridylyltransferase (EC 2.7.7.9) \|  \|  \|  \|  \| \| None \| Glycosyl transferase, family 2 \|  \|  \|  \|  \| \| None \| Glycosyl transferase, group 1 \|  \|  \|  \|  \| \| None \| Sugar-phosphate guanylyltransferase / Sugar-phosephate isomerase \|  \|  \|  \|  \| \| None \| Probable polysaccharide deacetylase pdaB precursor \|  \|  \|  \|  \| \| None \| PTS system, glucosamine-specific IIC component / PTS system, glucosamine-specific IIB component / PTS system, glucosamine-specific IIA component \|  \|  \|  \|  \| \| None \| Trehalose-6-phosphate hydrolase (EC 3.2.1.93) \|  \|  \|  \|  \| \| None \| PTS system, trehalose-specific IIB component (EC 2.7.1.201) / PTS system, trehalose-specific IIC component \|  \|  \|  \|  \| \| None \| Phosphoglycerate/bisphosphoglycerate mutase \|  \|  \|  \|  \| \| None \| carbohydrate kinase, FGGY( EC:2.7.1.17 ) \|  \|  \|  \|  \| \| None \| Rhamnulokinase (EC 2.7.1.5) \|  \|  \|  \|  \| \| None \| Maltodextrin ABC transporter, permease protein MdxG \|  \|  \|  \|  \| \| None \| Maltodextrin ABC transporter, ATP-binding protein MsmX \|  \|  \|  \|  \| \| None \| UDP-N-acetylglucosamine:L-malate glycosyltransferase \|  \|  \|  \|  \| \| None \| FIG013069: hypothetical protein co-occurring with TPR domain protein \|  \|  \|  \|  \| \| None \| FIG009300: TPR-repeat-containing protein \|  \|  \|  \|  \| \| None \| PTS system, maltose-specific IIC component / PTS system, maltose-specific IIB component (EC 2.7.1.208) \|  \|  \|  \|  \| \| None \| Maltose-6'-phosphate glucosidase (EC 3.2.1.122) \|  \|  \|  \|  \| \| None \| PTS system, glucose-specific IIA component \|  \|  \|  \|  \| \| None \| TPR repeat-containing protein YvcD \|  \|  \|  \|  \| \| None \| Undecaprenyl-phosphate alpha-N-acetylglucosaminyl 1-phosphate transferase (EC 2.7.8.33) \|  \|  \|  \|  \| \| None \| Fructose-1,6-bisphosphatase, GlpX type (EC 3.1.3.11) \|  \|  \|  \|  \| \| None \| Uncharacterized glycosyltransferase YwdF \|  \|  \|  \|  \| \| None \| GAF domain/HD domain protein \|  \|  \|  \|  \| \| None \| ABC transport protein, sugar-binding component yneA \|  \|  \|  \|  \| \| None \| 3-oxoacyl-[acyl-carrier protein] reductase paralog (EC 1.1.1.100) in cluster with unspecified monosaccharide transporter \|  \|  \|  \|  \| \| None \| Mannosyltransferase \|  \|  \|  \|  \| \| None \| D-galactose 1-dehydrogenase (EC 1.1.1.48) \|  \|  \|  \|  \| \| None \| PTS system, fructose-specific IIA component (EC 2.7.1.202) / PTS system, fructose-specific IIB component (EC 2.7.1.202) / PTS system, fructose-specific IIC component \|  \|  \|  \|  \| \| None \| Ferrous iron uptake system protein A \|  \|  \|  \|  \| \| None \| 1,2-diacylglycerol 3-glucosyltransferase (EC 2.4.1.157); diglucosyldiacylglycerol synthase (LTA membrane anchor synthesis) \|  \|  \|  \|  \| \| None \| Glucomannan utilization operon transcriptional regulator, GmuR \|  \|  \|  \|  \| \| None \| PTS system, oligo-beta-mannoside-specific IIC component \|  \|  \|  \|  \| \| None \| PTS system, oligo-beta-mannoside-specific IIA component (EC 2.7.1.205) \|  \|  \|  \|  \| \| None \| PTS system, oligo-beta-mannoside-specific IIB component (EC 2.7.1.205) \|  \|  \|  \|  \| \| None \| Endo-1,4-beta-xylanase (EC 3.2.1.8) \|  \|  \|  \|  \| \| None \| Xylan 1,4-beta-xylosidase (EC 3.2.1.37) \|  \|  \|  \|  \| \| None \| Xylan alpha-1,2-glucuronosidase (EC 3.2.1.131) \|  \|  \|  \|  \| \| None \| Predicted beta-xyloside ABC transporter, permease component \|  \|  \|  \|  \| \| None \| Putative sodium-glucose/galactose cotransporter \|  \|  \|  \|  \| \| None \| Predicted xylanase/chitin deacetylase \|  \|  \|  \|  \| \| None \| Maltose O-acetyltransferase (EC 2.3.1.79) \|  \|  \|  \|  \| \| None \| Glucokinase (EC 2.7.1.2) \|  \|  \|  \|  \| \| None \| Putative membrane peptidase, contains TPR repeat domain \|  \|  \|  \|  \| \| None \| TPR repeat-containing protein YrrB \|  \|  \|  \|  \| \| None \| TPR repeat protein \|  \|  \|  \|  \| \| None \| 6-phosphofructokinase (EC 2.7.1.11) \|  \|  \|  \|  \| \| None \| GGDEF domain protein \|  \|  \|  \|  \| \| None \| Lipopolysaccharide 1,2-N-acetylglucosaminetransferase (EC 2.4.1.56) \|  \|  \|  \|  \| \| None \| alpha-mannosidase (EC 3.2.1.24) \|  \|  \|  \|  \| \| None \| alpha-L-rhamnosidase (EC 3.2.1.40) \|  \|  \|  \|  \| \| None \| Predicted rhamnose oligosaccharide ABC transport system, permease component 2 \|  \|  \|  \|  \| \| None \| Predicted rhamnose oligosaccharide ABC transport system, permease component \|  \|  \|  \|  \| \| None \| Predicted rhamnose oligosaccharide ABC transport system, substrate-binding component \|  \|  \|  \|  \| \| None \| L-rhamnose isomerase (EC 5.3.1.14) \|  \|  \|  \|  \| \| None \| Predicted rhamnulose-1-phosphate aldolase (EC 4.1.2.19) / Predicted lactaldehyde dehydrogenase (EC 1.2.1.22) \|  \|  \|  \|  \| \| None \| Predicted L-rhamnose mutarotase \|  \|  \|  \|  \| \| None \| Predicted L-rhamnose permease RhaY \|  \|  \|  \|  \| \| None \| TPR domain protein, putative component of TonB system \|  \|  \|  \|  \| \| None \| alpha-L-arabinofuranosidase (EC 3.2.1.55) \|  \|  \|  \|  \| \| None \| Alpha-arabinosides ABC transport system, permease protein AraQ \|  \|  \|  \|  \| \| None \| Alpha-arabinosides ABC transport system, permease protein AraP \|  \|  \|  \|  \| \| None \| Alpha-arabinosides ABC transport system, substrate-binding protein AraN \|  \|  \|  \|  \| \| None \| L-arabinose ABC transporter, permease protein AraH \|  \|  \|  \|  \| \| None \| L-arabinose ABC transporter, ATP-binding protein AraG \|  \|  \|  \|  \| \| None \| L-arabinose ABC transporter, substrate-binding protein AraF \|  \|  \|  \|  \| \| None \| Response regulator for an arabinose sensor \|  \|  \|  \|  \| \| None \| Histidine kinase in an arabinose sensing sensor \|  \|  \|  \|  \| \| None \| Arabinose sensor protein \|  \|  \|  \|  \| \| None \| N-acetyl-D-glucosamine ABC transporter, permease protein 2 \|  \|  \|  \|  \| \| None \| PTS system, cellobiose-specific IIA component (EC 2.7.1.205) \|  \|  \|  \|  \| \| None \| PTS system, cellobiose-specific IIC component \|  \|  \|  \|  \| \| None \| PTS system, cellobiose-specific IIB component (EC 2.7.1.205) \|  \|  \|  \|  \| \| None \| PTS system, N-acetylglucosamine-specific IIC component / PTS system, N-acetylglucosamine-specific IIB component (EC 2.7.1.193) \|  \|  \|  \|  \| \| None \| Maltodextrin ABC transporter, substrate-binding protein MdxE \|  \|  \|  \|  \| \| None \| Maltodextrin ABC transporter, permease protein MdxF \|  \|  \|  \|  \| \| None \| UDP-galactose:(galactosyl) LPS alpha1,2-galactosyltransferase WaaW (EC 2.4.1.-) \|  \|  \|  \|  \| \| None \| Polysaccharide deacetylase, possible chitooligosaccharide deacetylase (EC 3.5.1.41) \|  \|  \|  \|  \| \| None \| alpha-galactosidase (EC 3.2.1.22) \|  \|  \|  \|  \| \| None \| beta-galactosidase (EC 3.2.1.23) \|  \|  \|  \|  \| \| None \| O antigen biosynthesis rhamnosyltransferase rfbN (EC 2.4.1.-) \|  \|  \|  \|  \| \| None \| PTS system, glucose-specific IIA component (EC 2.7.1.199) \|  \|  \|  \|  \| \| None \| PTS system, mannose-specific IIB component (EC 2.7.1.191) / PTS system, mannose-specific IIC component / PTS system, mannose-specific IIA component (EC 2.7.1.191) \|  \|  \|  \|  \| \| None \| PTS system, mannitol-specific IIC component / PTS system, mannitol-specific IIB component (EC 2.7.1.197) \|  \|  \|  \|  \| \| None \| Mannitol operon activator, BglG family \|  \|  \|  \|  \| \| None \| PTS system, mannitol-specific IIA component (EC 2.7.1.197) \|  \|  \|  \|  \| \| None \| Mannitol-1-phosphate 5-dehydrogenase (EC 1.1.1.17) \|  \|  \|  \|  \| \| None \| Mannosylglycerate hydrolase (EC 3.2.1.170) \|  \|  \|  \|  \| \| None \| PTS system, 2-O-alpha-mannosyl-D-glycerate-specific IIA component (EC 2.7.1.195) / PTS system, 2-O-alpha-mannosyl-D-glycerate-specific IIB component (EC 2.7.1.195) / PTS system, 2-O-alpha-mannosyl-D-glycerate-specific IIC component \|  \|  \|  \|  \| \| None \| PTS system, lactose/cellobiose specific IIB subunit \|  \|  \|  \|  \| \| None \| beta-N-acetylglucosaminidase (EC 3.2.1.52) \|  \|  \|  \|  \| \| None \| beta-glucosidase (EC 3.2.1.21) \|  \|  \|  \|  \| \| None \| 1,2-beta-oligoglucan phosphorylase (EC 2.4.1.333) \|  \|  \|  \|  \| \| None \| Sugar ABC transporter, permease protein precursor \|  \|  \|  \|  \| \| None \| Putative glycosyl transferase \|  \|  \|  \|  \| \| None \| Possible sporulation protein SpoIID precursor \|  \|  \|  \|  \| \| None \| Succinoglycan biosynthesis protein \| 0 \| 0 \|  \|  \| \| None \| Uncharacterized sugar epimerase YhfK \| 0 \| 0 \|  \|  \| \| None \| Spore coat polysaccharide biosynthesis protein SpsF \| 0 \| 0 \|  \|  \| \| None \| UDP-N-acetylglucosamine 4,6-dehydratase (inverting) (EC 4.2.1.115) \| 0 \| 0 \|  \|  \| \| None \| UDP-galactopyranose mutase (EC 5.4.99.9) \| 0 \| 0 \|  \|  \| \| None \| Glycerol-3-phosphate cytidylyltransferase (EC 2.7.7.39) \| 0 \| 0 \|  \|  \| \| None \| Glycosyltransferase (EC 2.4.1.-) \| 0 \| 0 \|  \|  \| \| None \| Lipopolysaccharide biosynthesis protein RffA \| 0 \| 0 \|  \|  \| \| None \| capsular polysaccharide biosynthesis protein \| 0 \| 0 \|  \|  \| \| None \| D-ribose pyranase (EC 5.4.99.62) \| 0 \| 0 \|  \|  \| \| None \| GGDEF/HD domain protein \| 0 \| 0 \|  \|  \| \| None \| Uncharacterized glycosyltransferase YcjM \| 0 \| 0 \|  \|  \| \| None \| N-acetylglucosaminyltransferase \| 0 \| 0 \|  \|  \| \| None \| Rhamnulose-1-phosphate aldolase (EC 4.1.2.19) \| 0 \| 0 \|  \|  \| \| None \| L-rhamnose mutarotase (EC 5.1.3.32) \| 0 \| 0 \|  \|  \| \| None \| PTS system, galactitol-specific IIA component (EC 2.7.1.200) \| 0 \| 0 \|  \|  \| \| None \| PTS system, galactitol-specific IIB component (EC 2.7.1.200) \| 0 \| 0 \|  \|  \| \| None \| PTS system, galactitol-specific IIC component \| 0 \| 0 \|  \|  \| \| None \| Galactitol-1-phosphate 5-dehydrogenase (EC 1.1.1.251) \| 0 \| 0 \|  \|  \| \| None \| Glycerol uptake operon antiterminator regulatory protein \| 0 \| 0 \|  \|  \| \| None \| PTS system, glucitol/sorbitol-specific IIA component (EC 2.7.1.198) \| 0 \| 0 \|  \|  \| \| None \| PTS system, glucitol/sorbitol-specific IIB component (EC 2.7.1.198) / PTS system, glucitol/sorbitol-specific IIC component 2 \| 0 \| 0 \|  \|  \| \| None \| PTS system, glucitol/sorbitol-specific IIC component \| 0 \| 0 \|  \|  \| \| None \| Sorbitol-6-phosphate 2-dehydrogenase (EC 1.1.1.140) \| 0 \| 0 \|  \|  \| \| Osmoregulation \| Glycerol uptake facilitator protein \|  \|  \|  \|  \| \| Sucrose utilization \| Fructokinase (EC 2.7.1.4) \|  \|  \|  \|  \| \| UDP-N-acetylmuramate from Fructose-6-phosphate Biosynthesis \| UDP-N-acetylenolpyruvoylglucosamine reductase (EC 1.1.1.158) \|  \|  \|  \|  \| \| UDP-N-acetylmuramate from Fructose-6-phosphate Biosynthesis \| Glucosamine-1-phosphate N-acetyltransferase (EC 2.3.1.157) \|  \|  \|  \|  \| \| UDP-N-acetylmuramate from Fructose-6-phosphate Biosynthesis \| UDP-N-acetylglucosamine 1-carboxyvinyltransferase (EC 2.5.1.7) \|  \|  \|  \|  \| \| UDP-N-acetylmuramate from Fructose-6-phosphate Biosynthesis \| Phosphoglucosamine mutase (EC 5.4.2.10) \|  \|  \|  \|  \| \| UDP-N-acetylmuramate from Fructose-6-phosphate Biosynthesis \| N-acetylglucosamine-1-phosphate uridyltransferase (EC 2.7.7.23) \|  \|  \|  \|  \| \| Xylose utilization \| Xylose ABC transporter, substrate-binding component \|  \|  \|  \|  \| \| Xylose utilization \| Xylose isomerase (EC 5.3.1.5) \|  \|  \|  \|  \| \| Xylose utilization \| Xylose ABC transporter, ATP-binding component \|  \|  \|  \|  \| \| Xylose utilization \| Xylulose kinase (EC 2.7.1.17) \|  \|  \|  \|  \| \| Xylose utilization \| Xylose-responsive transcription regulator, ROK family \|  \|  \|  \|  \| \| Xylose utilization \| Xylose ABC transporter, permease component \|  \|  \|  \|  \| \| Xylose utilization \| Xylose ABC transporter, permease protein XylH \|  \|  \|  \|  \| | | | | | | | | | | | | |
